# Advances and Challenges in the Breeding of Salt-Tolerant Rice

**DOI:** 10.3390/ijms21218385

**Published:** 2020-11-09

**Authors:** Hua Qin, Yuxiang Li, Rongfeng Huang

**Affiliations:** 1 Biotechnology Research Institute, Chinese Academy of Agricultural Sciences, Beijing 100081, China; qinhua@caas.cn (H.Q.); liyx0929@163.com (Y.L.); 2 National Key Facility of Crop Gene Resources and Genetic Improvement, Beijing 100081, China

**Keywords:** biotechnology breeding, high-throughput sequencing, QTLs, rice, salt tolerance

## Abstract

Soil salinization and a degraded ecological environment are challenging agricultural productivity and food security. Rice (*Oryza sativa*), the staple food of much of the world’s population, is categorized as a salt-susceptible crop. Improving the salt tolerance of rice would increase the potential of saline-alkali land and ensure food security. Salt tolerance is a complex quantitative trait. Biotechnological efforts to improve the salt tolerance of rice hinge on a detailed understanding of the molecular mechanisms underlying salt stress tolerance. In this review, we summarize progress in the breeding of salt-tolerant rice and in the mapping and cloning of genes and quantitative trait loci (QTLs) associated with salt tolerance in rice. Furthermore, we describe biotechnological tools that can be used to cultivate salt-tolerant rice, providing a reference for efforts aimed at rapidly and precisely cultivating salt-tolerance rice varieties.

## 1. Introduction

Plants grow in dynamic environments and frequently experience various abiotic stresses, such as drought, high salinity, cold, and heat [1]. Salt stress is one of the most severe environmental stresses. The effects of salt stress on plants include osmotic stress, ionic toxicity, and nutritional deficiencies and eventually lead to growth inhibition and crop yield losses [2,3]. Soil salinization is mainly caused by poor-quality drainage and irrigation systems, climate change (which leads to sea level rise), and drought [4]. Soil salinity is a global problem that affects more than 20% of cultivated land, including half of all irrigated areas, and this percentage is expected to increase [5]. Therefore, improving the salt tolerance of crops would not only lead to the effective use of saline-alkali land, but also support sustainable agriculture and alleviate the world food crisis. 

Rice (*Oryza sativa*) is an important staple food crop worldwide. As the global population continues to rise, rice production also needs to increase. However, global rice production is threatened by climate change [6]. Rice is considered to be a salt-susceptible species [7], and its salt tolerance depends on growth stage, organ type, and genotype [8,9,10]. Generally, the seedling and reproductive stages are more susceptible to salinity than the vegetative stage, roots are more sensitive than other organs [8], and *japonica* rice is more sensitive than *indica* rice [9]. Salinity stress suppresses photosynthesis and growth, leading to biomass loss, as well as partial sterility, which ultimately results in reductions in rice yield [11,12]. Therefore, the breeding of salt-tolerant rice cultivars is considered to be one of the most economic options to assure food security.

As a semi-aquatic plant, rice lives in a water-saturated environment during most of its life cycle. This environment has led to many distinct adaptations rice—for example, the ethylene response phenotype of rice is different from that of other species [13]. In *Arabidopsis thaliana*, ethylene plays a positive role in regulating salt tolerance, whereas, in rice, ethylene negatively regulates salt tolerance [14]. Since rice is an important food crop, yield and quality are two important criteria for rice producers. However, improving the stress tolerance of rice with less effect on yield and quality has been a challenge for breeders. It is hoped that the breeding of salt-tolerant rice varieties will be accelerated by dissecting the genetics underlying salt tolerance and using biotechnology to generate salt-tolerant plants. 

Several reviews have summarized the mechanisms of plant responses to salt stress [15,16,17], but only a few have focused on rice and the progress toward breeding salt-tolerant rice varieties. In this review, we summarize the current status of salt-tolerant rice breeding, recent advances in the mapping and cloning of salt-tolerant genes/quantitative trait loci (QTLs), and new technologies that can be used for breeding salt-tolerant rice varieties. We also highlight current challenges to breeding salt-tolerant rice, providing a basis for further studies and efforts aimed at breeding salt-tolerant rice varieties.

## 2. Salt-Tolerant Rice Identification and Evaluation Methods

The development of an efficient and reliable evaluation system is a prerequisite for breeding salt-tolerant rice varieties. The current rice salt tolerance indicators are divided into two aspects: morphological parameters and physiological parameters [18,19]. The morphological parameters evaluation method is to conduct salt treatments at different growth stages of rice and then observe and record the salt damage symptoms of plants, leaves, tillers, and spikelet fertility [20,21]. The standard evaluation score of visual salt injury was proposed by The International Rice Research Institute (IRRI); this method scores the salt tolerance of rice from 1 to 9 based on the tiller number, leaf symptoms, and the growth status of the whole plant under salt stress; the lower score (1) indicates tolerant and higher score (9) denotes sensitive genotypes [22]. However, this method is greatly affected by human qualitativeness, and there are time differences in the rate of leaf death and plant death of different materials. Therefore, this identification and evaluation system cannot completely and accurately reflect the salt tolerance of rice varieties.

Salinity stress induces metabolite changes, and several physiological mechanisms are perceived to contribute to the overall ability of rice plants to cope with excess salts [15,16,23]. Studies have shown that the Na^+^/K^+^ ratio, proline content, hydrogen peroxide, peroxidase activity, and sugars, etc. are affected under salt stress [21,24]. Therefore, it can be used to screen salt-tolerant rice varieties by comparing the changes of physiological and biochemical indices in rice with or without salt treatment. However, physiological and biochemical parameter methods lack specific evaluation standards, and the measurement of these indices requires corresponding instruments or kits, which are relatively cumbersome to operate.

Rice salt tolerance is a complex genetic and physiological characteristic, and the extent of its sensitivity varies during different growth and developmental stages [8,9,10]. The salt tolerance during the whole life of rice is a comprehensive reflection of the salt tolerance in each growth and developmental stage, which is closer to production practice and has more practical significance. However, due to soil heterogeneity, climatic factors and other environmental factors may influence the physiological processes; it is difficult to screen salt-tolerant rice varieties at the field level. Hence, screening under laboratory conditions is considered to be advantageous over field screening. Since the salt types in saline-alkali fields are double salts, the salt tolerance identified in the laboratory does not always correlate with that in the field. Therefore, the most reliable way to evaluate the salt tolerance of rice is to compare the changes of morphological parameters and physiological parameters in various growth and developmental stages under salt treatment and normal condition, both in the laboratory and in the field.

## 3. Salt Stress Affects Rice Growth and Grain Quality

The response and adaptation of rice to salt stress is a complex process. Salt stress causes root growth inhibition, leaf rolling, reduced plant height and tiller number, and spikelet sterility, which, ultimately, leads to a reduced yield [21,25]. In addition to morphological changes, salt stress also causes physiological and biochemical changes, such as the inhibition of photosynthesis; decreased water content; altered metabolism; increased Na transport to the shoot; and decreased K, Zn, and P uptake [12,25,26].

Since rice is a staple food, grain quality is an important driver of marketability. Rice grain is composed mainly of carbohydrates, predominantly starches. The determinants of rice grain quality are grain texture, taste, and visual attributes, which are further determined by the composition and structure of the starch molecules [27]. The effect of salt stress on rice grain starch depends on the salt concentration and the rice genotype. Specifically, salinity treatment leads to a decrease in starch content when the salt concentration is higher than 5-dS/m^2^ electrical conductivity, in both salt-tolerant and salt-susceptible cultivars of rice [28,29]. However, grain starch increased in the Nipponbar cultivar when low concentrations of salt (2 or 4-dS/m^2^ electrical conductivity) were applied at the anther appearance and seedling stages [30], suggesting that the response of rice to salt stress is complex and highly context-dependent.

Another indicator of rice grain quality is the nutritional value of the grain. Rice grain contains a variety of minerals, such as Ca, Mg, and P, and some trace elements, such as Fe, Cu, Zn, and Mn. Rice also contains the vitamins like thiamine, riboflavin, and niacin [25]. Several studies have shown that the absorption and uptake of micro- and macro-mineral nutrients are altered under salinity stress [31,32]. Saleethong et al. measured the macronutrients and micronutrients in grains of *Pokkali* (a salt-tolerant variety) and *KDML105* (a salt-sensitive variety) under saline conditions and found that salt stress resulted in significant reductions in macronutrient elements but an increase in Ca in brown rice grains of both cultivars. Moreover, the amounts of Mn, Cu, and Zn were higher in *Pokkali* than in *KDML105* [31]. Verma and Neue reported that the contents of Na, Fe, and Zn increased, while those of P and Mn decreased in rice grain with increased salinity, but the contents of N, Mg, Cu, K, and Ca were not affected in the varieties used in this study [32]. In addition to affecting the mineral content, salt stress can increase the rice grain protein content and amino acid levels [25]. Although little is known about the effects of salt stress on vitamins in rice grains, salinity caused a significant reduction in vitamin contents in wheat grains [33]. In summary, these studies show that the effect of salt stress on rice grain quality is multifaceted and depends on the salt concentration and the rice variety.

## 4. Breeding of Salt-Tolerant Rice Varieties

Developing elite salt-tolerant rice varieties is considered to be the most economically viable and environmentally friendly method to effectively use saline-alkali land. As a food crop, yield is an important indicator to evaluate the merits of rice varieties. Salt-alkali tolerance in rice is defined as the ability to grow on land with a 0.3% saline-alkali concentration, with a yield of more than 4500 kg per hectare. Through years of hard work, breeders have sought out, collected, evaluated, and developed many salt-tolerant rice resources.

Ceylon (modern-day Sri Lanka) was the first country to carry out the screening and cultivation of salt-tolerant rice varieties, introducing the first salt-tolerant rice variety, *Pokkali*, in 1939 [34]. Subsequently, India and the Philippines bred a series of salt-tolerant rice varieties, such as *Kala Rata 1-24*, *Nona Bokra*, *Bhura Rata*, *SR 26B*, *Chin.13*, and *349 Jhona*. Bangladesh bred *BRI*, *BR203-26-2*, *Sail*, and other salt-tolerant rice varieties. Thailand bred the salt-tolerant rice variety *FL530*. Japan bred the salt-tolerant rice varieties *Mantaro rice*, *Kanto 51*, *Hama Minoru*, *Chikushiqing*, and *Lansheng*. The United States bred the salt-tolerant rice variety *American Rice*. South Korea bred the salt-tolerant rice varieties *Dongjinbyeo*, *Ganchukbyeo*, *Gyehwabyeo*, *Ilpumbyeo*, *Seomjimbyeo*, and *Nonganbyeo*. Russia bred 16 the salt-tolerant rice varieties, including *VNIIR8207* and *Fontan* [35,36,37,38,39,40,41,42,43]. IRRI hosts more than 127,000 rice accessions collected worldwide, providing a rich source of genetic diversity. By evaluating the salt tolerance of these rice varieties, researchers identified approximately 103 varieties that were moderately to highly salt tolerant, including *Nona Bokra*, *Pamodar*, *Jhona349*, *IR4595-4-1-13*, *IR4630-22-2-5-1-3*, *IR9764-45-2-2*, and *IR9884-54-3* [44,45].

China began to study the salt tolerance of rice in the 1950s. In the 1980s, China launched a national collaborative research program on the salt-alkali resistance of rice and wheat. During the Seventh Five-Year Plan period (1986–1990), China began evaluating the salt tolerance of rice germplasm. This large-scale national cooperation resulted in some progress. The Liaoning Saline or Alkaline Land Utilization and Research Institute launched a salt-tolerant rice breeding program in the 1970s and cultivated a series of salt-tolerant *japonica* rice varieties, such as *Liaoyan No. 2*, *Liaoyan 241*, and *Liaoyan 16*. In 1984, the institute developed highly salt-tolerant *indica* rice varieties *81-210*. Since 1989, salt-tolerant varieties, such as *Salt-resistant No. 100*, *Yangeng 29*, *Yanfeng 47*, and *Yangeng 228*, have been cultivated [42]. The Jiangsu Institute of Agricultural Sciences in Coastal Areas began identifying and evaluating salt-tolerant rice germplasm resources in the 1980s. From more than 1300 germplasm resources, this group obtained 61 that were salt-tolerant [43], from which they developed many salt-tolerant materials, such as *Yancheng 156*, *Yandao No. 10*, and *Yandao No. 12* [43]. In addition, breeding institutes and breeders in China have used existing salt-tolerant germplasms or conventional breeding methods to obtain salt-tolerant rice varieties, such as *Changbai No. 6*, *Changbai No. 7*, *Changbai No. 9*, *Changbai No. 13*, *Jigeng No. 84*, and *Jinyuan 101* [42].

*Sea Rice 86* (*SR86*) is a new cultivar domesticated from a wild strain of rice that was first found in 1986 in saline-alkaline soil submerged in sea water near the coastal region of the city of Zhanjiang in Southeast China [46]. *SR86* showed a significantly higher ability to cope with high salinity than a highly salt-resistant rice variety, *Yanfen 47*, measured by both germination and salt inhibition rates [46]. After more than 20 years of breeding and selection, *SR86* retains an extraordinary tolerance to salinity and is considered to be a strategic germplasm resource for the development of new rice varieties. *SR86* is being used to investigate the mechanism of salt tolerance and effective breeding strategies. 

In recent years, our laboratory has also carried out a breeding program to develop salt-tolerant rice. We collected more than 750 rice accessions, including 500 rice accessions from all over the world and 250 salt-tolerant rice varieties from domestic coastal cities, such as Tianjin, Liaoning, Shandong, and Jiangsu. We investigated the salt tolerance of these varieties in Dongying, Shandong Province (37°31′29″ N 118°33′57″ E), a typical saline-alkali field in China. Briefly, thirty-day-old seedlings were transplanted to a normal field or saline field (0.35% NaCl, pH 8.2) at a spacing of 20 cm × 15 cm, and all agronomic traits were performed at the maturity stage. Through comparing the agronomic traits of the normal field and saline field, we selected a series of varieties with excellent agronomic traits and salt tolerance (Table 1) (unpublished data). Using these varieties, we made more than 300 hybrid combinations, generated more than 100 salt-tolerant genetic populations, and identified more than 1000 high-yielding salt-tolerant recombinant inbred lines.

## 5. Mapping and Cloning of Salt-Tolerant Genes/QTLs

Salt stress has two main stress effects on rice: osmotic stress and ionic stress [2,3]. Osmotic stress reduces the water uptake by roots and causes internal dehydration. It also leads to the excessive accumulation of reactive oxygen species (ROS), which damages various cellular components and macromolecules and, eventually, leads to plant death [47]. Ionic stress is caused by the excessive accumulation of Na^+^ and Cl^−^ in metabolically active intracellular compartments. High intracellular concentrations of Na^+^ inhibit the uptake of other ions, which can disrupt the metabolism and potentially kill the plant [2,3]. Thus, enhancing the ability of rice to minimize and adjust to osmotic and ionic stresses is an effective way to improve the salt tolerance of rice.

Salinity tolerance in rice is a polygenic trait controlled by QTLs [17]. Using mapping populations derived from crosses between salt-sensitive varieties and salt-tolerant varieties, researchers have identified a large number of QTLs (Table 2). Only a few major salt tolerance QTLs or genes have been identified by genomic methods. For example, *qSKC-1* and *qSNC-7* are involved in regulating K^+^/Na^+^ homeostasis under salt stress and explain 48.5% and 40.1%, respectively, of the total phenotypic variance [48]. Isolation of the *qSKC-1* gene by map-based cloning revealed that it encodes a member of the HKT-type transporter family [49]. The QTL *Saltol*, which acts to maintain shoot Na^+^/K^+^ homeostasis in the salt-tolerant cultivar *Pokkali*, explained 43% of the variation in the seedling shoot Na^+^/K^+^ ratio in a recombinant inbred line (RIL) population derived from a cross between *indica* varieties *IR29* and *Pokkali* [50]. *qSE3*, which encodes a K^+^ transporter gene, *OsHAK21*, promotes seed germination and seedling establishment under salinity stress in rice [51]. *qST1* and *qST3*, which are located on chromosomes 1 and 3, respectively, conferred salt tolerance at the young seedling stage and explained 36.9% of the total phenotypic variance in the RIL population derived from a cross between *Milyang 23* and *Gihobyeo* [52]. *qST1.1*, which plays a key role in salt tolerance in *SR86*, explained 62.6% of the phenotypic variance [53]. The identification of QTLs is only one step toward determining the genes associated with salinity tolerance; further research is needed to discover the major salt-tolerance genes and investigate their regulatory mechanisms in rice.

Recently, great progress has been made toward the identification and cloning of salt-tolerance genes through the molecular genetic analysis of plant responses to salt stress. For example, researchers have been interested in genes involved in ROS metabolism, because maintaining an appropriate level of ROS is essential for the survival of plants under salt stress. The level of ROS in plants depends on two processes: ROS biosynthesis and ROS scavenging. In rice, a salt treatment induces or represses the expression of *respiratory burst oxidase homologs* (*Rbohs*), which catalyze the conversion of O_2_ to O_2_^−^ [71], suggesting that *Rbohs* are candidate genes for improving salt tolerance in rice. In addition to the genes involved in ROS biosynthesis, genes related to ROS scavenging also have the potential to improve rice salt tolerance. For example, ascorbate peroxidase (APX) catalyzes the conversion of H_2_O_2_ to H_2_O and O_2_ using ascorbate as the specific electron donor [72]. The overexpression of *APX* genes in rice reduces ROS levels and enhances salt tolerance [73,74,75]. The knockdown of *OsVTC1-1* and *OsVTC1-3*, which are involved in ascorbate synthesis, increases the accumulation of ROS and decreases the salt resistance of rice [76,77,78].

Genes involved in Na^+^/K^+^ homeostasis are also candidates for improving salt tolerance in rice. High-salinity stress results in altered K^+^/Na^+^ ratios, which lead to metabolic changes in the plant [79]. Na^+^ causes growth inhibition via Na^+^ toxicity, whereas K^+^ is essential for plant growth and development. Therefore, restricting the intracellular accumulation of toxic sodium (Na^+^) is beneficial for survival under salt stress [2,3]. Members of the high-affinity K^+^ transporter (HKT) family in rice function as Na^+^ and Na^+^/K^+^ transporters in controlling Na^+^ accumulation, and enhancing or inhibiting the expression of *HKT* genes changes the intracellular Na^+^ content and the salt tolerance of rice [80,81]. Thus, modifying ion channels is an option to improve the salt tolerance of rice.

In addition to genes related to ROS and Na^+^/K^+^ homeostasis, many other genes influence the salt tolerance of rice [16]. For example, *rice expansin 7* (*OsEXPA7*), which encodes a cell wall-loosening protein, positively regulates salt tolerance in rice by coordinating sodium transport, ROS scavenging, and cell-wall loosening [82]. *Response regulator 22* (*OsRR22*) encodes a B-type response regulator protein that acts as a transcription factor regulating genes involved in the osmotic stress response and/or ion transport between parenchyma cells and vascular tissue cells in the root [83]. OsCYP71D8L, a cytochrome P450 monooxygenase in the CYP71 clan in rice, negatively regulates salt tolerance by affecting gibberellin and cytokinin homeostasis [84]. The dominant suppressor of KAR2 (OsDSK2a) is a ubiquitin-binding protein that mediates seedling growth and salt responses in rice through interacting with elongated uppermost internode (EUI) to affect gibberellin metabolism [85]. Although a number of salt-tolerance genes have been identified (Table 3), further information is needed about how to effectively use these genes to improve rice salt tolerance.

## 6. Biotechnology Promises to Accelerate Breeding of Salt-Tolerant Rice Cultivars

Publication of the rice reference genome and the development of next-generation sequencing (NGS) techniques provide an opportunity to explore genome-wide genetic variations and carry out genotyping in a highly efficient way [86]. The relatively low cost of sequencing enables the use of genome and transcriptome sequencing to discover a large number of sequence polymorphisms and to map some agronomic traits [87]. Genome-wide association studies (GWAS) are a powerful approach for identifying valuable natural variations in trait-associated loci, as well as allelic variations in candidate genes underlying quantitative and complex traits, including those related to growth, salt tolerance, and nutritional quality [88,89]. Compared to a traditional QTL linkage analysis, GWAS is based on high-density variations in natural populations and can detect multiple alleles at the same site [90]. Several loci associated with salt tolerance have been identified in rice based on GWAS. Kumar et al. conducted a GWAS of 12 different salt tolerance-related traits at the reproductive stage and identified 20 single-nucleotide polymorphisms (SNPs) significantly associated with the Na^+^/K^+^ ratio and 44 SNPs associated with other traits observed under salt stress conditions [91]. Liu et al. used 708 rice accessions to perform a GWAS to identify the genes associated with rice salt tolerance and identified seven accessions carrying favorable haplotypes of four genes significantly associated with grain yield under salt stress. These promising candidates will provide valuable resources for salt-tolerant rice breeding [89]. Lekklar et al. conducted a GWAS for salt tolerance during rice reproduction, and more than 73% of the identified loci overlapped with the previously reported salt QTLs [92]. Yuan et al. performed a GWAS using 664 cultivated rice accessions from the 3000 Rice genomes, and twenty-one salt-tolerant QTLs and two candidate genes were identified [93]. These studies indicate that GWAS is a powerful strategy for mapping QTLs of salt tolerance in rice.

Effective phenotyping data (phenomics data) is a prerequisite for the discovery of genes/QTLs, association mapping, and GWAS. Despite recent advances in genomics, the lack of appropriate phenomics data limits the progress in genomics-assisted crop improvement programs. Therefore, the acquisition of high-throughput, effective, and comprehensive trait data in rice has become an acute need [94,95]. High-throughput phenotyping offers the opportunity capture phenotypically complex variations underpinning adaptation in traditional phenotypic selection or statistics-based breeding programs [94,95]. With the development of plant phenotyping platforms, we can obtain the phenomics data more effectively and cost-efficiently, and the integration of high-throughput trait phenotyping with genomics will greatly promote the genetic dissection of salt tolerance-related traits.

Genomic and transcriptomic analysis, proteomics, and metabolomics are powerful tools for identifying genes related to salt tolerance in rice. Salt stress causes a series of changes in rice, including changes in gene expression, protein content, and metabolite levels [96,97,98]. By comparing the transcriptome, proteome, and metabolome of rice under salt stress versus normal conditions, or in salt-tolerant versus salt-sensitive varieties, we can obtain many potential genes related to salt tolerance. For example, a study comparing transcriptome profiles of *FL478* and *IR29* found more than one thousand genes that were differentially expressed under salt stress compared to normal conditions [96]. Sun et al. analyzed the transcriptome data of a salt-tolerant rice landrace called *Changmaogu* and detected a large number of genes that were differentially expressed at the germination and seedling stages under salt stress. A further analysis revealed that most of the differentially expressed genes were clustered in the pathways of ABA signal transduction and carotenoid biosynthesis [97]. Peng et al. used an Isobaric Tags for Relative and Absolute Quantitation (iTRAQ)-based proteomic technique to detect proteins that become more or less abundant under salt stress and identified 332 differentially abundant proteins in seedlings of *salinity-tolerant and dwarf 58* (*sd58*) and *Kitaake* [98]. Although these studies provide many candidate genes for salinity tolerance, whether these genes can be used for breeding salt-tolerant rice remains to be verified. The transgenic approach and genome editing approach are powerful tools for verifying whether genes can be used for salt-tolerant rice breeding. Using the transgenic approach and genome editing approach, ideal materials for target genes can rapidly be obtained and used to assess the salt tolerance, which would accelerate efforts to improve the salinity tolerance in rice.

Based on high-density genome-wide SNP markers detected by next-generation sequencing, the SNP marker-assisted selection method is used to accelerate the process of breeding salt-tolerant rice. Using this method, Rana et al. precisely introgressed the *hitomebore salt tolerant 1* (*hst1*) gene from the salt-tolerant cultivar *Kaijin* into the high-yielding cultivar *Yukinko-mai*. Their offspring, *YNU31-2-4*, had agronomic traits similar to *Yukinko-mai* under normal growth conditions. Under salt stress, the yield of *YNU31-2-4* was significantly higher than that of *Yukinko-mai* [99]; Bimpong et al. used marker-assisted selection to develop salt-tolerant rice cultivars through introgressing *Saltol* into the lowland cultivar, *Rassi*, and obtained 16 introgression lines for further African-wide testing prior to release in six West African countries [100]. Pauyawaew et al. introgressed *Saltol* into *KDML105* by two rounds of marker-assisted backcrossing, and introgression lines with positive *Saltol* alleles are being tested for salinity tolerance in the salt-affected areas in the northeast of Thailand [101]. All these results suggest that SNP marker-assisted selection breeding is a fast and effective method to improve rice salt tolerance. 

The most current methods of genomic selection use single SNP markers to predict the genetic merits of individuals, but haplotypes may have several advantages over single markers for genomic selection. A haplotype is the combination of a series of genetic mutations that coexist on a single chromosome; it contains multiple SNPs and can better capture the linkage disequilibrium and genomic similarity in different lines [102,103]. The use of haplotypes can improve the accuracy of genomic prediction. Thus, identifying key haplotypes related to the salt tolerance of rice will provide useful genetic resources and parent materials for breeding salt-tolerant rice. 

## 7. Challenges and Perspectives

Salt stress is one of the main environmental factors affecting rice growth and rice yield worldwide. Improving the salt tolerance of rice is the most direct and effective method to solve this problem. After years of collection and screening, scientists have obtained several salt-tolerant rice germplasms (Table 2), which have laid the foundation for studying the mechanism of salt tolerance in rice and developing salt-tolerant rice cultivars. This review summarizes the progress to date on the breeding of salt-tolerant rice and proposes that genomics and molecular tools for precision breeding will accelerate the development of salt-tolerant cultivars.

Rice salt tolerance is controlled by multiple genes and is a complex physiological characteristic [17]. The evaluation of rice salt tolerance is also complex. Phenotypic traits are typically used to evaluate the salt tolerance of rice varieties [18], an approach that lacks comprehensiveness and accuracy and may result in an assessment of salt tolerance that is not consistent with actual performances in the field. Therefore, a standardized system to evaluate rice salt tolerance is urgently needed. Moreover, salt tolerance is genotype-dependent. Although numerous QTLs controlling salt tolerance traits have been identified in different mapping populations (Table 2), only a few major salt tolerance genes have been isolated from QTLs [48,49]. The inconsistency and variability of QTLs in different genetic backgrounds and environments have limited their applications in breeding programs. 

Using traditional breeding methods, scientists have bred a series of salt-tolerant rice cultivars, but the mechanism of salt tolerance in rice is largely unclear. How plants perceive salt stress, how these signals are translated into adaptive responses, and how multiple salt tolerance genes coordinately regulate salt tolerance in rice are all questions that need further investigation. Moreover, traditional breeding methods are time-consuming and inefficient. SNP marker-assisted selection and genetic engineering technology will greatly improve the molecular breeding process [99,104]. Therefore, efforts should be made to capture useful salt tolerance genes as possible genetic markers to introgress into elite rice varieties. In addition, it is difficult to obtain salt-tolerant varieties that can be used in field production by the introduction of a single gene or several genes. To cultivate valuable salt-tolerant rice varieties, multiple salt tolerance genes or haplotypes need to be gathered into elite rice varieties; how to quickly and effectively introduce these genes or haplotypes simultaneously is another problem that needs to be solved. With the innovation of breeding technology, many new breeding technologies, such as MutMap, knock-out/in, and genome editing, will accelerate the process of salt-tolerant rice cultivation [83,105]. Further studies should focus on cloning salt tolerance genes and elucidating their regulatory mechanisms and on investigating how multiple genes or haplotypes can be transferred at the same time with stable inheritance by their offspring.

Due to variations in saline-alkali soils and environmental conditions in different areas, the cultivation of salt-tolerant rice varieties is subject to strong geographical constraints; varieties cultivated in one place are unsuitable for planting in another. Therefore, research efforts should focus on identifying molecular markers and haplotypes associated with salt tolerance and on developing salt-tolerant rice. The main rice varieties in various regions should be transformed with genes associated with improved salt tolerance by using a combination of molecular breeding and traditional breeding approaches. The resulting core rice germplasm with a high salt tolerance and excellent agronomic traits would provide a valuable germplasm resource for breeding programs aimed at developing salt-tolerant rice.

## Figures and Tables

**Table 1:** Rice varieties with excellent agronomic traits and salt tolerance results.

Material Code	Plant Height (cm)		Panicle Length (cm)		Tiller Number		Yield (kg/hectare)	
	Control	Salt	Control	Salt	Control	Salt	Control	Salt
DYST1	94.6 ± 1.8	73.1 ± 3.5	21.6 ± 0.7	19.4 ± 1.0	8.0 ± 1.2	5.6 ± 0.5	6438.0	6271.5
DYST2	92.8 ± 3.2	71.8 ± 3.0	18.2 ± 1.8	16.9 ± 1.2	10.6 ± 1.5	9.6 ± 0.9	7159.5	5106.0
DYST3	91.3 ± 4.1	64.9 ± 4.2	19.2 ± 1.6	17.4 ± 1.5	13.0 ± 0.7	7.2 ± 1.1	8325.0	4662.0
DYST4	91.5 ± 3.9	78.8 ± 4.0	19.2 ± 1.1	18.4 ± 1.8	11.4 ± 1.8	8.0 ± 2.0	7270.5	6216.0
DYST5	99.7 ± 2.6	83.3 ± 1.8	22.8 ± 1.3	20.3 ± 1.6	11.8 ± 1.8	8.8 ± 2.2	8325.0	5217.0
DYST6	95.6 ± 1.0	74.6 ± 3.6	21.0 ± 0.6	18.9 ± 0.9	13.2 ± 0.8	11.0 ± 2.9	8103.0	5050.5
DYST7	91.6 ± 2.6	79.4 ± 5.4	21.8 ± 1.3	20.5 ± 0.7	11.8 ± 2.2	10.6 ± 1.7	6993.0	5050.5
DYST8	96.0 ± 3.1	81.5 ± 3.0	21.3 ± 0.7	20.3 ± 1.3	12.0 ± 1.4	10.8 ± 2.3	7992.0	5827.5
DYST9	95.6 ± 2.8	80.6 ± 3.0	21.3 ± 1.2	20.6 ± 1.1	11.6 ± 1.8	10.2 ± 2.9	8268.8	5142.0
DYST10	95.2 ± 1.8	83.0 ± 1.0	20.4 ± 1.6	19.7 ± 1.4	11.2 ± 2.4	10.2 ± 1.6	8880.0	5550.0

“Control” indicates that the variety was grown in a normal field. “Salt” indicates that the variety was grown in a field containing 0.35% NaCl and pH 8.2 throughout its life cycle. “DYST” means Dongying Salt Tolerance.

**Table 2:** Quantitative trait loci (QTLs) identified for salt tolerance in rice.

Parents	Number of QTLs	Stage	Reference
Capsule × BRRI dhan29	30	seedling	[54]
DJ15 × Koshihikari	9	seedling	[55]
Hasawi × IR29	20	seedling	[56]
CSR27 × MI48	25	seedling, vegetative and reproductive	[57]
Xiushui09 × IR2061-520-6-9	47	seedling	[58]
At354 × Bg352	6	seedling	[59]
Pokkali × Bengal	50	seedling	[60]
Cheriviruppu8 × Pusa Basmati 1	16	reproductive	[61]
Teqing × Oryza rufipogon Griff	15	seedling	[62]
Milyang 23 × Gihobyeo	2	seedling	[52]
Nona Bokra × Koshihikari	11	seedling	[48]
CSR27 × MI48	8	maturity	[63]
Nona Bokra × Jupiter	33	seedling	[64]
IR75862 × Ce258/Zhongguangxiang1	18	seedling	[65]
Pokkali × IR36	6	maturity	[49]
Tarommahali × Khazar	2	seedling	[66]
Jiucaiqing × IR26	22	seedling	[67]
Jiucaiqing × IR26	16	germination	[68]
9311 × Oryza rufipogon Griff	10	seedling	[69]
Dongnong425 × Changbai10	13	seedling	[70]
Pokkali × IR29	17	seedling	[50]

**Table 3:** Genes associated with rice salt tolerance.

Genes Name	Accession Number	Gene Function
*OsSOS1*	Os12g0641100	Exports Na^+^ ions out of cells, positively regulates salt tolerance
*OsHKT1;1*	Os04g0607500	Mediate Na^+^-specific transport, positively regulates salt tolerance
*OsHKT1;4*	Os04g0607600	Mediate Na^+^-specific transport, positively regulates salt tolerance
*OsHAK21*	Os03g0576200	K^+^ transporter, positively regulates salt tolerance
*OsEIN2*	Os07g0155600	Ethylene signaling component, negatively regulates salt tolerance
*OsEIL1*	Os03g0324300	Ethylene signaling component, negatively regulates salt tolerance
*OsEIL2*	Os07g0685700	Ethylene signaling component, negatively regulates salt tolerance
*OsAPX2*	Os07g0694700	Encoding ascorbate peroxidases, positively regulates salt tolerance
*OsCPK12*	Os04g0560600	Encoding a calcium-dependent protein kinase, positively regulates salt tolerance
*DST*	Os03g0786400	Encoding Zinc-finger protein, negatively regulates salt tolerance
*SIT1*	Os02g0640500	Encoding a lectin receptor-like kinase, positively regulates salt tolerance
*OsDOF15*	Os03g0764900	Encoding a DOF-binding with one finger transcription factor, negatively regulates salt tolerance
*OsTIR1*	Os05g0150500	Auxin receptor, positively regulates salt tolerance
*OsAFB2*	Os04g0395600	Auxin receptor, positively regulates salt tolerance
*OsGA2ox5*	Os07g0103500	Encoding a gibberellin metabolism enzyme, positively regulates salt tolerance
*OsTIR1*	Os05g0150500	Auxin receptor, positively regulates salt tolerance
*DRO1*	Os07g0614400	Associated with root angle modifications, negatively regulates salt tolerance
*OsEXPA7*	Os03g0822000	Encoding cell wall-loosening proteins, positively regulates salt tolerance
*OsPIL14*	Os07g0143200	Phytochrome interacting factor like gene, positively regulates salt tolerance
*SLR1*	Os03g0707600	rice DELLA protein, GA signaling suppressor, positively regulates salt tolerance
*OsDSK2a*	Os03g0131300	The UBL-UBA protein, negatively regulates salt tolerance
*IDS1*	Os03g0818800	Encoding an apetala2/ethylene response factor transcription factor, negatively regulates salt tolerance
*AGO2*	Os04g0615700	Encoding a ARGONAUTE family protein, positively regulates salt tolerance
*BG3*	Os01g0680200	A putative cytokinin transporter, positively regulates salt tolerance
*OsRR2*	Os06g0183100	Encoding a B-type response regulator, negatively regulates salt tolerance
*OsRR9*	Os11g0143300	Negative regulators of cytokinin signaling, negatively regulates salt tolerance
*OsRR10*	Os12g0139400	Negative regulators of cytokinin signaling, negatively regulates salt tolerance
*OsC2DP*	Os09g0571200	Encoding a novel C2 domain-containing protein, positively regulates salt tolerance
*OsOTS1*	Os06g0487900	The ubiquitin-like protease class of SUMO protease, positively regulates salt tolerance
*OsZFP179*	Os01g0839100	Encoding C2H2-type zinc-finger protein, positively regulates salt tolerance
*OsZFP182*	Os03g0820300	Encoding TFIIIA-type zinc-finger protein, positively regulates salt tolerance
*OsZFP185*	Os02g0195600	Encoding A20/AN1-type zinc-finger protein, negatively regulates salt tolerance
*OsZFP213*	Os12g0617000	Encoding C2H2-type zinc-finger protein, positively regulates salt tolerance
*OsZFP245*	Os07g0587400	Encoding TFIIIA-type zinc-finger protein, positively regulates salt tolerance
*OsZFP252*	Os12g0583700	Encoding TFIIIA-type zinc-finger protein, positively regulates salt tolerance
*OsMGT1*	Os01g0869200	A rice Mg2+ transporter, positively regulates salt tolerance
*CYP71D8L*	Os02g0184900	Encoding a cytochrome P450 monooxygenases, positively regulates salt tolerance
*OsCYP94C2b*	Os12g0150200	Encoding a JA-catabolizing enzyme, positively regulates salt tolerance
*OsVTC1-1*	Os01g0847200	GDP-D-mannose pyrophosphorylase (GMPase) catalyzes the synthesis of GDP-D-mannose, positively regulates salt tolerance
*OsVTC1-3*	Os03g0268400	GDP-D-mannose pyrophosphorylase (GMPase) catalyzes the synthesis of GDP-D-mannose, positively regulates salt tolerance
